# A ^90^Y-labelled anti-ROBO1 monoclonal antibody exhibits antitumour activity against hepatocellular carcinoma xenografts during ROBO1-targeted radioimmunotherapy

**DOI:** 10.1186/s13550-014-0029-3

**Published:** 2014-06-01

**Authors:** Kentaro Fujiwara, Keitaro Koyama, Kosuke Suga, Masako Ikemura, Yasutaka Saito, Akihiro Hino, Hiroko Iwanari, Osamu Kusano-Arai, Kenichi Mitsui, Hiroyuki Kasahara, Masashi Fukayama, Tatsuhiko Kodama, Takao Hamakubo, Toshimitsu Momose

**Affiliations:** 1 Department of Radiology, Graduate School of Medicine, The University of Tokyo, 3-1, Hongo 7-Chome, Bunkyo-ku 113-8655, Tokyo, Japan; 2 SANKYO LABO SERVICE Co., Ltd., 2-13-16, Nishiichinoe, Edogawaku 132-0023, Tokyo, Japan; 3 Department of Pathology, Graduate School of Medicine, The University of Tokyo, 3-1, Hongo 7-Chome, Bunkyo-ku 113-8655, Tokyo, Japan; 4 FUJIFILM RI Pharma Co., Ltd., 453-1, Shimo-Okura, Matsuo-Machi, Sammu-City 289-1592, Chiba, Japan; 5 Department of Quantitative Biology and Medicine, Research Center for Advanced Science and Technology, The University of Tokyo, 4-6-1 Komaba, Meguro-ku 153-8904, Tokyo, Japan; 6 Institute of Immunology Co., Ltd., 1-1-1 Koraku, Bunkyo 112-0004, Tokyo, Japan; 7 Department of Systems Biology and Medicine, Research Center for Advanced Science and Technology, The University of Tokyo, 4-6-1 Komaba, Meguro-ku 153-8904, Tokyo, Japan

**Keywords:** Hepatocellular carcinoma, Radioimmunotherapy, ROBO1, 90Y, Biodistribution

## Abstract

**Background:**

ROBO1 is a membrane protein that functions in axon guidance. ROBO1 contributes to tumour metastasis and angiogenesis and may have potential as a target protein of immunotherapy because ROBO1 is specifically expressed at high levels in hepatocellular carcinoma. In this study, we examined biodistribution and radioimmunotherapy (RIT) using a radioisotope-labelled anti-ROBO1 monoclonal antibody (MAb) against hepatocellular carcinoma models.

**Methods:**

ROBO1-positive HepG2 human hepatocellular carcinoma xenograft nude mice were used in this study. We conjugated anti-ROBO1 MAb with 1,4,7,10-tetraazacyclododecane-1,4,7,10-tetraacetic acid (DOTA), and the conjugates were labelled with ^111^In and ^90^Y. To study biodistribution, the ^111^In-DOTA-anti-ROBO1 MAb was injected into HepG2 xenograft mice *via* the tail vein. To evaluate any antitumour effect, a RIT study was performed, and the ^90^Y-DOTA-anti-ROBO1 MAb was injected *via* the tail vein. Tumour volume, mouse weight, and blood cell count were periodically measured throughout the experiments. The tumours and organs of mice were collected, and a histopathological analysis was carried out.

**Results:**

The tumour uptake of ^111^In-anti-ROBO1 MAb in HepG2 xenograft mice was 15.0% ± 0.69% injected dose per gram at 48 h after injection.

Immunotherapy with cold-anti-ROBO1 MAb (70 μg) did not cause a significant antitumour effect. RIT with 6.7 MBq of ^90^Y-anti-ROBO1 MAb caused significant tumour growth suppression. Transient body weight loss and bone-marrow suppression were observed. Histopathological analyses of tumours revealed the fatal degeneration of tumour cells, significant reduction of the Ki-67 index, and an increase of the apoptosis index. Normal organs showed no significant injury, but a transient reduction of hematopoietic cells was observed in the spleen and in the sternal bone marrow.

**Conclusions:**

These results suggest that RIT with ^90^Y-anti-ROBO1 MAb is a promising treatment for ROBO1-positive hepatocellular carcinoma.

## Background

Hepatocellular carcinoma (HCC) is a malignant tumour derived from hepatocytes. Primary liver cancer is the third most common cause of death from cancer worldwide, and patients have a poor prognosis [1,2]. The primary treatments for HCC are surgical resection, transplantation, and chemoembolisation. Sorafenib, an oral multikinase inhibitor, is the standard medicine administered for advanced HCC. However, Sorafenib treatment does not result in remission or improved survival [1]. Systemic chemotherapy has no survival benefit [1,3]. Therefore, the development of more effective therapeutic drugs for HCC is required.

Radioimmunotherapy (RIT) is a type of radiotherapy using a radio-labelled monoclonal antibody (MAb) that targets a tumour-specific antigen [4-6]. RIT can attack targeted antigen-positive tumour cells and also antigen-negative tumour cells by the cross-fire effect [6,7]. Although Zevalin and Bexxar have been successfully used to treat relapsed or refractory non-Hodgkin's lymphomas, the success of RIT as treatment for solid tumours has been limited, due to the radioresistance of solid tumours and an inability to deliver a sufficient dose to bulky tumours without bone-marrow toxicity [4,6,8]. However, RIT has the potential to be therapeutically efficacious for metastasis as well as primary lesion [6].

The human homologue of the *Drosophila* roundabout gene, *ROBO1*, encodes an axon guidance receptor that is classified in a novel subfamily of the immunoglobulin (Ig) superfamily and is highly conserved in species ranging from fruit flies to mammals [9,10]. Previous reports indicate that ROBO1 contributes to tumour metastasis and angiogenesis [11-13]. In fact, ROBO1 is up-regulated in 85% of HCC cases [14]. Therefore, ROBO1 has potential as a target protein of RIT for HCC.

In this study, the pharmacokinetics of an ^111^In (half-life 2.8 days)-labelled anti-ROBO1 MAb (^111^In-anti-ROBO1) was investigated *in vivo* by a biodistribution study. Then, RIT using a ^90^Y (half-life 2.7 days)-labelled anti-ROBO1 MAb (^90^Y-anti-ROBO1) was carried out to evaluate antitumour activity and the effect of radiation exposure on normal organs, with respect to pathology. In this report, we demonstrate antitumour effects of ^90^Y-anti-ROBO1 on xenograft tumours in nude mice.

## Methods

### Cell culture and animal models

A HepG2 human HCC cell line was purchased from Health Science Research Resources Bank (Osaka, Japan). Male BALB/c nude mice (4 to 5 weeks of age) were purchased from CLEA Japan Inc. (Tokyo, Japan). HepG2 cells were grown in DMEM and cultured in a medium supplemented with 10% (*v*/*v*) foetal bovine serum at 37°C in a humidified atmosphere with 5% CO_2_. For the HepG2 tumour-bearing animal models, the HepG2 cells (2 × 10^6^, 200 μL) were inoculated subcutaneously into the right flanks of male BALB/c nude mice. At 5 weeks after inoculation, the mice were subjected to the studies. All animal studies were approved by the Animal Care Committee of The University of Tokyo.

Sf9 cells were cultured in Grace's supplemented media (Invitrogen) containing 10% foetal calf serum, as described [15].

### Antibodies

A MAb against human ROBO1 was generated as previously described [14,15]. Briefly, human *ROBO1* cDNA was polymerase chain reaction (PCR)-amplified from Alexander cells and inserted into the pBlueBac 4.5-TOPO vector. The recombinant baculovirus expressing *ROBO1* was immunized directly into *gp64* transgenic mice. A positive hybridoma clone, B5209B, was selected by the reactivity to the ROBO1 stable cell line, by flow cytometry. An anti-hemagglutinin (HA) antibody was purchased from Sigma (St. Louis, MO, USA). MAb B5209B was purified by ammonium sulphate precipitation from the ascitic fluid of nude mice, to which the hybridoma cells were implanted intraperitoneally. To raise a MAb, which recognizes cell surface ROBO1, *gp64* transgenic mice were immunized subcutaneously with 1 mg of ROBO1-expressing budded baculovirus with *pertussis* toxin adjuvant, as previously described [15].

### Evaluation of ROBO1-binding affinity of the anti-ROBO1 antibody

To evaluate binding affinities of the MAb against ROBO1, we prepared a stable ROBO1-expressing cell line and a soluble form of the ROBO1 (sROBO) protein.

The sROBO protein was affinity-purified from the culture supernatant of Sf9 cells infected with recombinant baculoviruses, which harboured a gene fragment encoding the extracellular domain of the human ROBO1 (1-861 aa) protein with V5 and 6 × His tags at its C-terminus.

A CHO cell line stably expressing ROBO1 fused with an HA tag (ROBO1-HA) was generated using the Flp-In System (Life Technologies Japan Corp., Tokyo, Japan). *ROBO1* fused to *HA*, encoding the tag at the C-terminal, was inserted into the pcDNA5/FRT vector and was co-transfected with the pOG44 vector to Flp-In-CHO cells using Lipofectamine 2000. A highly ROBO1-HA-expressing clone was selected from the 1 mg/mL hygromycin-resistant cells.

Specificity and affinity of the anti-ROBO1 antibody, B5209B, were evaluated using flow cytometry or cell ELISA, as previously described [15,16]. For flow cytometry, wild type or ROBO1-HA-expressing Flp-In-CHO cells were incubated with primary antibodies for 1 h at a concentration of 1 μg/mL. For the anti-HA antibody, 0.02% saponin was added for permeabilisation. The cells were then washed with dilution buffer (1% bovine serum albumin and 0.1 mM EDTA in PBS) and reacted with R-Phycoerythrin conjugated anti-mouse IgG (Jackson ImmunoResearch Laboratories, West Grove, PA, USA) diluted to 1:200 with dilution buffer. Finally, cells were washed twice with dilution buffer and analysed by flow cytometry (GUAVA EasyCyte™ Plus System; Millipore, Billerica, MA, USA).

**Figure 1:**
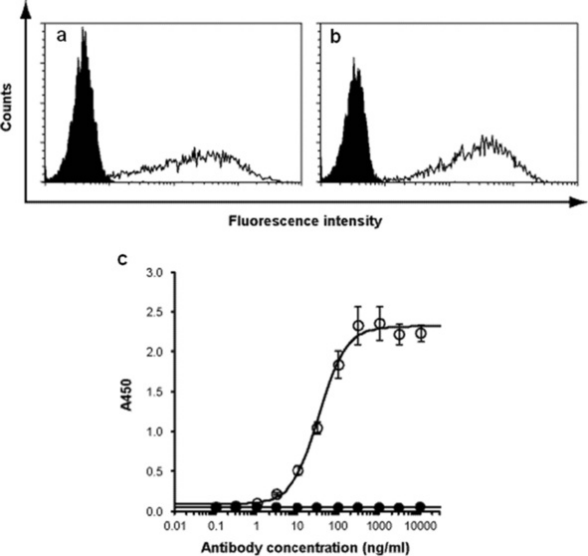
**Reactivity of anti-ROBO1 MAb.** Specific binding of **(a)** anti-HA antibody and **(b)** anti-ROBO1 IgG (filled histogram, wild type CHO-cells; open histogram, ROBO1-HA-expressing Flp-In-CHO cells) by flow cytometry. **(c)** Dose-dependent reactivity of the anti-ROBO1 IgG by cell ELISA (filled circle, wild type CHO-cells; open circle, ROBO1-HA-expressing Flp-In-CHO cells).

For the cell ELISA, 10^5^ cells/well were plated on poly-d-lysine-coated, 96-well plates. The plates were centrifuged at 2,000 rpm for 1 min, and supernatants were discarded. Then, the plates were blocked with blocking buffer [40% Block Ace (Dainippon Sumitomo Pharma, Osaka, Japan) in 10 mM Tris-buffered saline (TBS)] for 30 min. Cells were incubated with primary antibody of various concentrations in blocking buffer for 1 h. After washing twice with 0.05% Tween 20 in saline, cells were incubated for 30 min with peroxidase-conjugated anti-mouse IgG Fc-specific (Jackson ImmunoResearch). After washing three times, the enzymatic reaction was visualized with TMB Soluble Reagent (ScyTek Laboratories, Logan, UT, USA). After the reaction was stopped with TMB Stop Buffer (ScyTek Laboratories), the absorbance was measured at 450 nm using a microplate reader (Biotrak II; GE Healthcare, Piscataway, NJ, USA). The data were fitted with a four parameter logistic curve (Figure 1c).

### DOTA conjugation and radiolabelling

1,4,7,10-tetraazacyclododecane-1,4,7,10-tetraacetic acid (DOTA) was purchased from Macrocyclics (Dallas, TX, USA). ^111^InCl_3_ was obtained from Nordion Inc. (Vancouver, Canada). ^90^YCl_3_ was obtained from Eckert and Ziegler (Braunschweig, Germany).

The anti-ROBO1 MAb in 0.1 M NaHCO_3_ buffer (pH 9.0) was conjugated to DOTA at a molar ratio of 1:10 (protein to chelate). After incubation for 1 h at 37°C, the MAb-chelate was purified on an ultra-filtration column (ultra-4; Millipore, Billerica, MA, USA).

^90^YCl_3_ was added to the DOTA-anti-ROBO1 MAb in 0.25 M ammonium acetate buffer (pH 5.5). The mixture was incubated for 1 h at 45°C. The ^90^Y-DOTA-anti-ROBO1 MAb (^90^Y-anti-ROBO1) was purified on a desalting column equilibrated with PBS (NAP-5 column; GE Healthcare, Buckinghamshire, UK).

Labelling yield and radiochemical purity were estimated by instant thin layer chromatography (Pall Corp., MI, USA). A similar procedure was used to prepare the ^111^In-DOTA-anti-ROBO1 MAb (^111^In-anti-ROBO1).

Competitive ELISA was implemented to assess potency of the anti-ROBO1 MAb, DOTA-anti-ROBO1 MAb, and ^90^Y- and ^111^In-anti-ROBO1 MAb. sROBO was coated onto 96-well assay plates, and then plates were blocked by Tris buffer containing 1% BSA. The serially diluted antibody solutions were mixed with horse radish peroxidase (HRP)-labelled anti-ROBO1 MAb (HRP-anti-ROBO1; The University of Tokyo). Aliquots of the mixed solution were added to each well of the assay plate and incubated for 2 h at 37°C. The assay plate was washed with TBS-T, and TMB solution was added to each well and incubated for 5 min at room temperature. Absorbance was measured at 450-nm wavelength with a microplate reader (SpectraMax; Molecular Devices, Sunnyvale, CA, USA). IC_50_ was calculated using GraphPad Prism4 software (GraphPad; San Diego, CA, USA).

### Biodistribution study

HepG2 xenograft (858.3 ± 237 mm^3^) mice were randomly divided into six groups (*n* = 3 per group). Each mouse was injected with 0.37 MBq of ^111^In-anti-ROBO1 (10 μg) *via* the tail vein. The mice were euthanised at 6, 24, 48, 72, 144, and 240 h after injection. Blood, heart, lung, liver, kidney, spleen, stomach, intestine, muscle, femoral bone, sternum, and tumour were collected, weighed, and measured for radioactivity. The percentage of injected dose per gram of tissue (% ID/g) was calculated for each organ.

### RIT and immunotherapy

HepG2 xenograft mice were randomly divided into three groups (*n* = 4 to 5 per group). Mice were injected *via* the tail vein with a single dose of 6.7 MBq of either ^90^Y-anti-ROBO1 (70 μg, *n* = 5 mice), cold-anti-ROBO1 MAb (70 μg, *n* = 4 mice), or saline (*n* = 5 mice). Tumour volumes for the mice in the ^90^Y-anti-ROBO1, cold-anti-ROBO1, and saline groups were 674.9 ± 315, 545.0 ± 342, and 437.6 ± 223 mm^3^, respectively.

The tumour size, body weight, and blood cell count were measured twice a week until day 28 after injection. The tumour volume was calculated using the following formula: 0.5 × (shortest diameter)^2^ × (longest diameter). Tumour growth (%) was calculated using the formula: (tumour volume at each time point)/(tumour volume on day0) × 100. Peripheral blood was collected from the tail vein and then tested in an automated haematology analyser, Celltac α (MEK-6400; Nihon Kohden, Tokyo, Japan).

Mice were euthanised when the tumour size was >1,500 mm^3^ or the body weight decreased by >20% of its original weight.

### Histological analysis

Tissue specimens of HepG2 tumour, liver, kidney, intestine, spleen, femoral bone, and sternum were obtained from mice at day 0, 7, 14, and 28 after injection of ^90^Y-anti-ROBO1 (*n* = 3 per group). All samples were fixed in 4% paraformaldehyde overnight at 4°C. They were embedded in paraffin, and 3 to 5 μm sections were obtained. The femoral bone and sternum samples were decalcified before embedding in paraffin. Slide sections, excluding the femoral bone and sternum, were deparaffinised, dehydrated, and stained with haematoxylin and eosin (H&E). Femoral bone and sternum sections were stained with Giemsa solution.

Ki-67 staining and terminal deoxynucleotidyl transferase-mediated dUTP nick-end labelling (TUNEL) were carried out on HepG2 sections to investigate the status of tumour cell proliferation and apoptosis, respectively.

For Ki-67 stain, antigen retrieval was performed in 10 mM citrate buffer solution (pH 6.0) in a pressure cooker for 15 min. Endogenous peroxidase activity was quenched using 0.3% hydrogen peroxide-methanol for 30 min, and then the specimens were blocked with 5% normal goat serum for 1 h. Specimens were incubated with Ki-67 MAb (1:100; DAKO, Carpinteria, CA, USA) overnight at 4°C. Next, the specimens were treated with a secondary antibody (simple Stain MAX-PO; Nichirei, Tokyo, Japan) for 30 min. To visualize peroxidase activity, 0.2 mg/mL 3,3′-diaminobenzidine (Dojindo Laboratories, Kumamoto, Japan) was used as the substrate in 0.05 M Tris-HCl buffer (pH 7.6) containing 0.01% H_2_O_2_. Nuclear staining was achieved with haematoxylin.

Apoptosis was detected using ApopTag Plus Peroxidase In Situ Apoptosis Detection Kit (Chemicon International, Inc., Billerica, MA, USA) for TUNEL, per the manufacturer's instructions.

Proliferative and apoptotic cells were quantified by determining the percentage of Ki-67-positive and TUNEL-positive cells, respectively. Two regions of interest measuring 200 × 200 μm were established on each tumour per one section. The percentage of positive-stained cells among all the counted tumour cells was calculated.

### Statistical analysis

Data are expressed as the mean ± standard deviation (SD). Statistical analyses were carried out using JMP Pro 9.0.3 software. Means were compared using one-way ANOVA and Student's *t* test. *p* values <0.05 were considered statistically significant.

## Results

The following are the gathered results:

1. ROBO1-binding affinity of the anti-ROBO1 MAb

The ROBO1 binding activity of the selected hybridoma clone, B5209B, was evaluated using ROBO1-expressing CHO cells. MAb B5209B exhibited specific binding to ROBO1-expressing CHO cells, as compared to the positive control anti-HA antibody (Figure 1a,b). A dose-dependent binding study to ROBO1-expressing cells showed a saturating response curve. The apparent half-maximal binding to cell surface ROBO1 was estimated to be approximately 32.5 ng/mL from the fitted curve in ELISA (Figure 1c).

2. DOTA conjugation and radiolabelling

Labelling yields and radiochemical purification of ^90^Y- and ^111^In-anti-ROBO1 were greater than 99%. Competitive ELISA revealed that anti-ROBO1, DOTA-anti-ROBO1, and ^90^Y- and ^111^In-anti-ROBO1 inhibited the binding of HRP-anti-ROBO1 to the sROBO1 antigen, in a dose-dependent manner. IC_50_ values for anti-ROBO1, DOTA-anti-ROBO1, and ^90^Y-anti-ROBO1 were 0.47, 0.41, and 0.51 μg/mL, respectively. Similarly, IC_50_ values for anti-ROBO1, DOTA-anti-ROBO1, and ^111^In-anti-ROBO1 were 0.41, 0.44, and 0.60 μg/mL, respectively. These results indicate that the DOTA-anti-ROBO1 and ^90^Y- and ^111^In-anti-ROBO1 possess similar potency as that of the anti-ROBO1.

3. Biodistribution of ^111^In-anti-ROBO1 MAb

**Figure 2:**
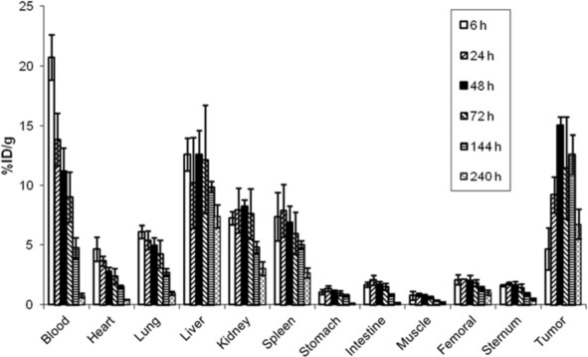
**Biodistribution of**^**111**^**In-anti-ROBO1 in HepG2 xenograft mice.** Results represent the calculated percentages of the injected dose per gram of tissue (% ID/g).

The biodistribution study using ^111^In-anti-ROBO1 was carried out using HepG2 xenograft nude mice (Figure 2). The tumour uptake of ^111^In-anti-ROBO1 was 4.68 ± 1.8, 9.22 ± 1.5, 15.0 ± 0.69, 11.5 ± 4.3, 12.6 ± 1.6, and 6.73 ± 1.1% ID/g at 6, 24, 48, 72, 144, and 240 h after injection, respectively. The maximal tumour uptake of ^111^In-anti-ROBO1 occurred at 48 h after injection and then decreased slowly.

High retention of tracer in the blood was observed with 20.7% ± 1.9% ID/g at 6 h but decreased to 9.03 ± 2.1% ID/g at 72 h. ^111^In-anti-ROBO1 showed high uptake in the liver with 12.6 ± 2.0% ID/g at 48 h and then decreased to 9.9 ± 0.5% ID/g at 144 h.

Kidney and spleen uptake of ^111^In-anti-ROBO1 was 8.24 ± 0.51% ID/g at 48 h and 7.87 ± 2.2% ID/g at 24 h, respectively.

In the sternum, the maximal uptake of ^111^In-anti-ROBO1 was 1.80 ± 0.1% ID/g, and in the femoral bone, it was 2.14 ± 0.005% ID/g at 24 h.

4. RIT and immunotherapy

**Figure 3:**
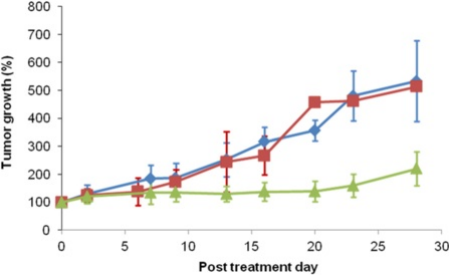
**Antitumour effect of**^**90**^**Y-anti-ROBO1 in mouse models.** Tumour growth (%) (diamonds, saline; squares, cold-anti-ROBO1; triangles, ^90^Y anti-ROBO1).

The therapeutic efficacy of ^90^Y-anti-ROBO1 was investigated in HepG2 xenograft mice (Figure 3). The tumours in the ^90^Y-anti-ROBO1 group showed significant growth inhibition, compared with growth in the saline and cold-anti-ROBO1 groups from day 13 to 20 (*p* < 0.05); however, tumour regrowth was observed at day 23. The cold-anti-ROBO1 did not show any significant anti-tumour effect compared with the saline treatment.

**Figure 4:**
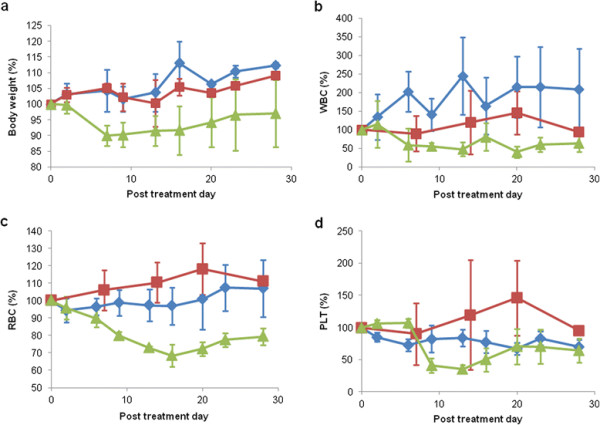
**Effect of**^**90**^**Y-anti-ROBO1 on body weight and blood counts in mouse models. (a)** Mean body weight of HepG2 xenograft mice. **(b,c,d)** Blood cell count of HepG2 xenograft mice (diamonds, saline; squares, cold-anti-ROBO1; triangles, ^90^Y anti-ROBO1).

Animal body weight was measured after injection (Figure 4a). Although the average body weight decreased to about 90% of the original value in the ^90^Y-anti-ROBO1 group, the change was transient. Other mice injected with saline or cold-anti-ROBO1 showed no significant reduction in body weight.

White blood cell (WBC), red blood cell (RBC), and platelet (PLT) counts were measured after injection (Figure 4b,c,d). The ^90^Y-anti-ROBO1 group showed a reduction of WBC count to 40.3% ± 14.8% of the original value. RBC and PLT counts of the ^90^Y-anti-ROBO1 group were reduced to 68.4% ± 6.4% and 35.4% ± 6.4% of the original values, respectively. Blood cell counts of the ^90^Y-anti-ROBO1 group decreased significantly compared with those of the saline and cold-anti-ROBO1 groups.

**Figure 5:**
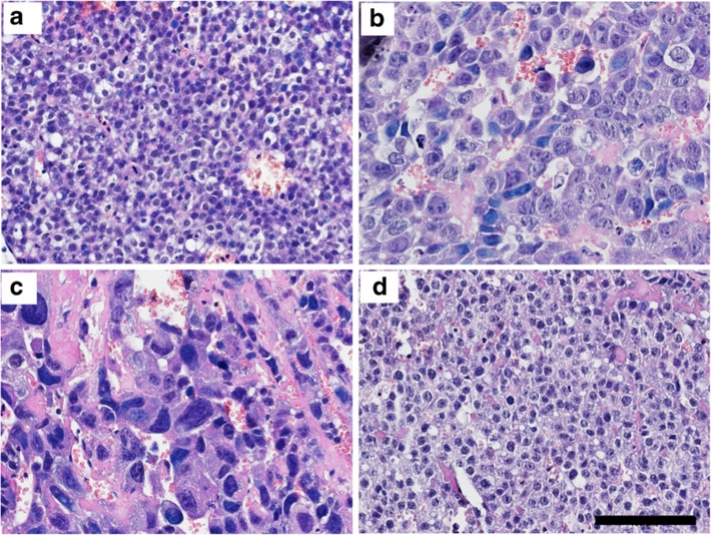
**Histological analysis of tumours. (a)** HepG2 tumour on day 0, original magnification × 400; **(b)** HepG2 tumour on day 7, original magnification × 400; **(c)** HepG2 tumour on day 14, original magnification × 400; **(d)** HepG2 tumour on day 28, original magnification × 400. Scale bar = 100 μm.

We performed H&E staining using paraffin sections of resected tissues. Tumour cell degeneration, determined by cell body swelling, chromatin compaction, and apoptotic cells, was observed on day 7 and day 14 post injection (Figure 5b,c). However, tumour cell degeneration decreased on day 28. Pathological characteristics of tumour cells, such as shape and size, were not altered and appeared to be the same as those observed at day 0 (Figure 5a,d).

**Figure 6:**
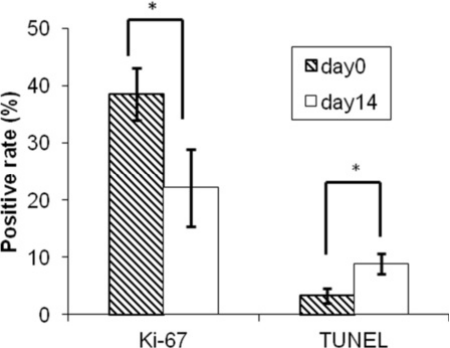
**Quantitative analysis of cell proliferation and apoptosis.** Ki-67: ratio of Ki-67-positive cells; TUNEL: ratio of TUNEL-positive cells. *p < 0.01

The Ki-67 analysis showed a reduction of the proliferative index in the ^90^Y-anti-ROBO1 group at day 14 (22.2% ± 6.8%) compared with that at day 0 (38.5% ± 4.5%, *p* < 0.05; Figure 6).

The percentage of TUNEL-positive cells on day 14 significantly increased (8.82% ± 1.8%) compared with that at day 0 (3.28% ± 1.3%, *p* < 0.01; Figure 6).

No apparent pathological changes were observed in the liver, kidney, or intestine (data not shown).

**Figure 7:**
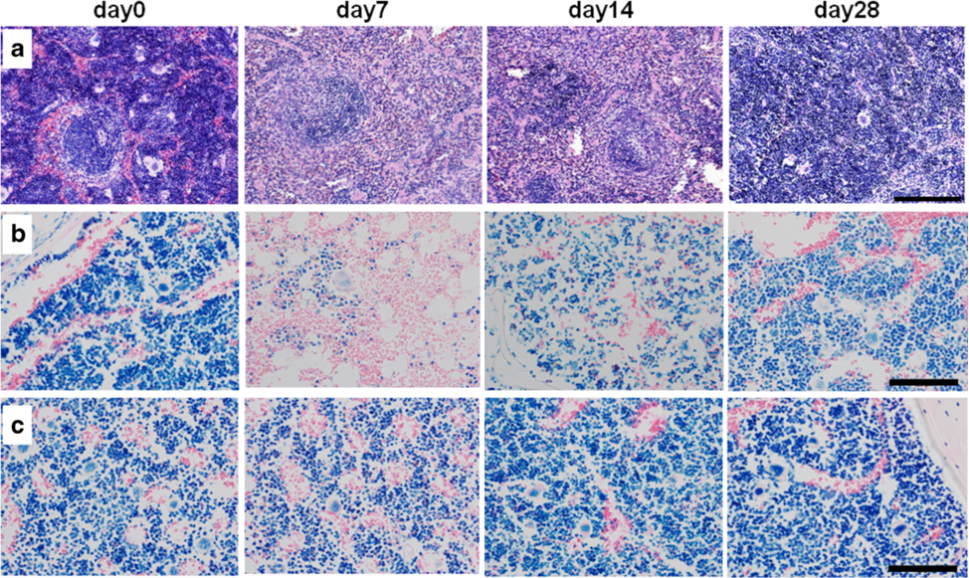
**Histological analysis of the spleen, sternum, and femoral bone. (a)** Spleen, scale bar = 200 μm. **(b)** Sternum, scale bar = 100 μm. **(c)** Femoral bone, scale bar = 100 μm.

On day 7, the spleen showed a remarkable decrease of hematopoietic cells in the red pulp (Figure 7a). Regenerating hematopoietic foci of erythroid and granulocytic cells were observed in 14 days. On day 28, regenerating megacaryocytes were observed. Hematopoietic cells in the red pulp drastically increased and comprised a large portion of the spleen.

We performed Giemsa staining of the sternum and femoral bone (Figure 7b,c). On day 7, a remarkable decrease of hematopoietic cells was observed in sternum bone marrow. Hematopoietic cell count was reduced to about 20% to 50%, compared with control count. The cell count recovered to approximately 80% on day 28. In contrast, no decrease of hematopoietic cells was observed in the femoral bone marrow.

## Discussion

The results of this study are the first to demonstrate that ^111^In-anti-ROBO1 can target ROBO1 and that ^90^Y-anti-ROBO1 has significant antitumour effects against HCC xenografts.

To evaluate the ROBO1-binding affinity of the anti-ROBO1, we performed cell ELISA. Anti-ROBO1 exhibited high half-maximal binding (32.5 ng/mL) to cell surface ROBO1 on ROBO1-expressing CHO cells. Then, we performed competitive ELISA to evaluate the potency of anti-ROBO1, DOTA-anti-ROBO1, and radioisotope (RI)-labelled anti-ROBO1. The *in vitro* analysis confirmed that DOTA conjugation and radiolabelling of anti-ROBO1 did not impair the receptor affinity for anti-ROBO1. Therefore, it is reasonable to presume that anti-ROBO1 can be used as an RI-labelled antibody.

We used ^111^In-anti-ROBO1 as a biodistribution surrogate to predict *in vivo* behaviours of ^90^Y-anti-ROBO1, because previous reports indicate that ^111^In- and ^90^Y-labelled antibodies, proteins, and peptides are biologically equivalent with respect to their uptake in tumours and other major organs [17].

The results from the biodistribution studies show that the uptake of ^111^In-anti-ROBO1 in HepG2 xenografts was higher than that in unaffected organs. The tumour uptake of radiotracer reached a maximum of 15.0% ± 0.69% ID/g at 48 h after injection and remained high for an extended period of time (12.6% ± 1.6% ID/g at 144 h after injection). The uptake of radiotracer to unaffected organs showed a maximum of 12.6% ± 2.1% in the liver at 48 h, 8.24% ± 0.51% ID/g in the kidney at 48 h, and 7.87% ± 2.2% ID/g in the spleen at 24 h after injection, but the uptake of each organ decreased gradually with time. These results are similar to those reported in previous studies for RI-labelled IgGs specific for other antigens [18-20]. Therefore, the results confirm that ^111^In-anti-ROBO1 specifically targets ROBO1, and it is expected that ^90^Y-anti-ROBO1 would accumulate in ROBO1-positive tumours because of the similar binding specificity.

We injected 6.7 MBq of ^90^Y-anti-ROBO1 into HepG2 xenograft mice. ^90^Y-anti-ROBO1 significantly suppressed tumour growth, compared with saline and cold-anti-ROBO1 treatment. There was no significant difference in tumour growth between the saline and cold-anti-ROBO1 groups. These findings reveal that cold-anti-ROBO1 has no significant antitumour effect.

^90^Y-anti-ROBO1 showed significant tumour growth suppression, indicated by the measurement of tumour volume, and we observed pathological changes, such as cell degeneration and an increase in apoptotic cells. In previous studies of RIT, the treated tumour showed pathological changes, such as necrosis, apoptosis, fibrosis, and a decrease of Ki-67-positive cells [18-21]. Our pathological analysis showed an increase of apoptotic cells and a decrease of Ki-67-positive cells, without necrosis or fibrosis. In contrast, cell degeneration, cell body swelling, and chromatin compaction were observed. Cell degeneration is a common pathological change in treated tumour tissues. Thus, our pathological analysis supports the conclusion that ^90^Y-anti-ROBO1 possesses significant antitumour effects when used in RIT.

Unfortunately, we observed tumour regrowth at about day 20. In the pathological study, a decrease of cell degeneration was observed at day 28; furthermore, the shape and size of tumour cells appeared to be the same as those observed at day 0. These results suggest that the antitumour effect was insufficient to completely inhibit tumour growth. This phenomenon might be attributed to the radioresistance of solid tumours. Solid tumours require a higher radiation dose for a complete response compared with haematological malignancies, which are radiosensitive [22]. Tumour cell hypoxia is one of the main factors causing radioresistance [23]. In this study, it is possible that hypoxic cells in the tumours were induced, because the tumour volumes of the mice were relatively large. Thus, tumour size might limit the antitumour effects of RIT. Therefore, the injection of ^90^Y-anti-ROBO1 in combination with a radiosensitiser to small tumours may deliver more effective antitumour activity [24].

Body weight loss and an anaemic condition caused by pancytopenia were observed. These conditions were transient, and recovery was evident at the end of the observation period. No histopathological changes were observed in the normal organs, but changes were observed in the spleen and sternum bone marrow. Therefore, systemic toxicity of ^90^Y-anti-ROBO1 was transient and limited to hematopoietic systems.

We observed a difference in the degree of damage between the sternum and femur, although the biodistribution study showed no significant difference of ^111^In-anti-ROBO1 accumulation between the two. These contradictory results might be explained by the radiation exposure from blood circulation and high accumulation areas. Tumours in the right flank and liver showed high accumulation of ^111^In-anti-ROBO1. In addition, the cardiac pool was also a high accumulation area because of the remaining of ^111^In-anti-ROBO1 in the blood. The sternum is physically closer to these areas compared to the femur; consequently, the different proximities are the reason for the disparity observed in damage between the two. Esteban et al. reported that radiation exposure to the hematopoietic system can originate from either circulating ^90^Y-MAb conjugate or non-specifically, from nearby ^90^Y-MAb bound to a tumour [25,26]. Taken together, the observations in this study and previous reports suggest that the radiation exposure from a high accumulation area is responsible for the damage to hematopoietic tissues.

In this study, we evaluated the antitumour effects and side effects of ^90^Y-anti-ROBO1 for an injection dose of 6.7 MBq. We determined the injection dose of 6.7 MBq, based on an injection dose of 7.4 MBq that provided maximal antitumour efficacy without animal mortality [21]. The side effects, such as bone marrow suppression and animal mortality, are the main limiting factors of dose-escalation for enhancement of the therapeutic effect. In the toxicity study, we observed that the damage to organs was transient and limited to the hematopoietic systems. Hence, our data suggest that it might be possible to administer a higher dose of ^90^Y-anti-ROBO1. A dose-escalation study is needed to determine a maximal tolerated dose.

## Conclusion

We evaluated a radiolabelled anti-ROBO1 IgG as a possible candidate for RIT of HCC. Based on the biodistribution study, administration of ^111^In-anti-ROBO1 demonstrated a high accumulation to HepG2 xenografts, which represent the human HCC model. Moreover, administration of ^90^Y-anti-ROBO1 demonstrated antitumour activity towards HepG2 xenografts, which was confirmed by a pathological study. In conclusion, our data suggest that administration of ^90^Y-anti-ROBO1, which targets ROBO1, might become an effective tool for RIT treatment of HCC and can be applied to other ROBO1-positive cancers.

## Competing interests

The authors declare that they have no competing interests.

## Authors' contributions

KF, KK, KS, TK, and TM designed the study. KF, KK, KS, and TM performed the animal study and drafted the manuscript. HI, OKA, KM, and TH developed and evaluated the antibody. YS, AH, and HK performed DOTA conjugation and radiorabelling of the antibody. KF, MI, and MF performed pathological analysis. All authors read and approved the final manuscript.
